# Parent and practitioner experiences of opt-out consent in neonatal intensive care: a mixed methods study within a trial

**DOI:** 10.1136/archdischild-2025-328693

**Published:** 2025-08-31

**Authors:** Tracy Mitchell, Izabela Andrzejewska, Cheryl Battersby, Christina Cole, Zoe Daskalopoulou, Jon Dorling, Chris Gale, Michaela Graham, Marie Hubbard, Pollyanna Hardy, Madeleine Hurd, Andrew Robert King, Brett James Manley, David Murray, Elizabeth Nuthall, Heather O’Connor, Shalini Ojha, Calum T Roberts, Amy Rodriquez, Charles Christoph Roehr, Kayleigh Stanbury, Lyvonne Tume, Lauren Young, Kerry Woolfall

**Affiliations:** 1 Public Health, Policy and Systems, University of Liverpool, Liverpool, UK; 2 Chelsea and Westminster Hospital NHS Foundation Trust, London, UK; 3 School of Public Health and Centre for Paediatrics and Child Health, Imperial College London, London, UK; 4 National Perinatal Epidemiology Unit, Nuffield Department of Population Health, Oxford, UK; 5 Department of Neonatal Medicine, University Hospital Southampton NHS Foundation Trust, Southampton, UK; 6 Academic Neonatal Medicine, Imperial College London, London, UK; 7 Leicester Royal Infirmary, University Hospitals of Leicester, Leicester, UK; 8 Department of Obstetrics, Gynaecology and Newborn Health, The University of Melbourne, Melbourne, Victoria, Australia; 9 The Royal Women’s Hospital, Melbourne, Victoria, Australia; 10 School of Medicine, University of Nottingham, Derby, UK; 11 Neonatal Unit, University Hospitals of Derby and Burton NHS Foundation Trust, Derby, UK; 12 Department of Paediatrics, Monash University Faculty of Medicine, Nursing and Health Sciences, Clayton, Victoria, Australia; 13 Monash Newborn, Monash Children’s Hospital, Clayton, Victoria, Australia; 14 Monash Newborn, Monash Health, Clayton, Victoria, Australia; 15 Nursing and Midwifery, La Trobe University, Bundoora, Victoria, Australia; 16 Faculty of Health and Life Sciences, The University of Bristol, Bristol, UK; 17 Faculty of Health, Social Care & Medicine, Edge Hill University, Ormskirk, UK; 18 Alder Hey Children’s NHS Trust, Liverpool, UK; 19 Patient and Public Involvement Partner, Support for the Sick Newborn and Their Parents (SSNAP), Oxford, UK

**Keywords:** Neonatology, Qualitative research, Ethics, Intensive Care Units, Neonatal

## Abstract

**Background:**

In neonatal trials, verbal opt-out consent has been used to reduce burden on families and make recruitment more efficient and representative. It involves information provision through posters and leaflets before randomisation, and parents can verbally ‘opt out’ of their baby being randomised to the trial. There is limited understanding of how opt-out consent is operationalised in a multicentre neonatal trial, and its acceptability to staff and parents.

**Objective:**

To explore views and experiences of verbal opt-out consent in neoGASTRIC, a neonatal randomised trial comparing routine and no routine measurements of gastric contents in preterm babies.

**Methods:**

A mixed methods (questionnaires, interviews and focus groups) process evaluation within a trial.

**Setting:**

Four UK neonatal units.

**Participants:**

253 participants: 167 staff (149 questionnaires; 18 across two focus groups), 86 parents (85 questionnaires; 15 interviews; 14 took part in both).

**Results:**

Parents and staff supported opt-out consent in neoGASTRIC as interventions were viewed as low risk and non-invasive. Parents appreciated an appropriately timed research conversation; only 21% noticed study information banners/posters. Operationalisation of opt-out consent varied in terms of when information was provided and randomisation timing. Women approached during labour or within hours of birth reported feeling overwhelmed and lacking capacity to consider research. Some staff operationalised a modified opt-in approach.

**Conclusions:**

An appropriately timed verbal opt-out approach to consent was seen acceptable as proportionate in the neonatal context in a low-risk trial comparing different accepted clinical, non-pharmaceutical, practices. Findings informed neoGASTRIC and will guide approaches to consent in this setting.

**WHAT IS ALREADY KNOWN ON THIS TOPIC**

- Previous research has questioned the validity of informed consent for neonatal trials where fear, anxiety and time pressure may compromise informed decision-making.
- Alternatives to informed consent include an ‘opt-out’ approach which may address such concerns.
- There is a paucity of data describing how a verbal opt-out approach is experienced by parents and operationalised by staff in neonatal trials.

**WHAT THIS STUDY ADDS**

- Parents and staff viewed an appropriately timed verbal opt-out approach to consent as acceptable in a low-risk, non-pharmaceutical, neonatal comparative effectiveness trial.
- There was variance in how verbal opt-out consent was operationalised, with some staff using a verbal opt-in approach.
- Some parents approached during labour or within hours of birth felt that they lacked capacity to consider research participation.
- A minority of parents recalled seeing the prominently placed posters and banners highlighting the need for individual-level participant information provision.

**HOW THIS STUDY MIGHT AFFECT RESEARCH, PRACTICE OR POLICY**

- Future neonatal trials should consider and evaluate use of a research without prior consent approach for time-critical interventions.
- Where a verbal opt-out approach to consent is used, staff training and guidance should emphasise appropriately timed written and verbal information provision prior to randomisation.

## Introduction

 Appropriately powered and methodologically robust clinical trials are needed to provide evidence-based care for critically ill babies. The challenges of seeking informed consent from parents for their babies’ inclusion in a neonatal trial are well documented.^1^ Several studies have questioned the validity of informed consent when fear, anxiety and time pressure may compromise voluntariness and decision-making capacity.^2^ A systematic review of ethical issues regarding consent to neonatal trials^3^ included 30 studies and concluded that none of the consent processes used were satisfactory due to parental incapacity to provide informed consent. They highlighted alternative approaches to consent in neonatal trials, such as emergency consent and ‘assent’ for participation.

Several international neonatal randomised trials have used variations of an ‘opt-out’ approach to consent when assessing low-risk comparative effectiveness interventions^4^ to reduce burden and improve recruitment.^5 6^ Such trials involve automatic trial enrolment as the default after parents have been provided the opportunity to ‘opt out’ of their baby’s involvement. These studies have involved interventions used in clinical practice (eg, withholding milk feeds around blood transfusion^4^) that do not fall under emergency legislation for research without prior consent (RWPC) as there is time (eg, >48 hours) between admission and randomisation. There is an ethical debate^7^ about whether opt-out approaches respect an individual’s right to decide on research involvement; evident in inconsistency between UK Research Ethics Committee’s decisions regarding the validity of opt-out consent.^8^ There is variance in how opt-out consent is operationalised, with one pilot trial^9^ finding that some staff operationalise verbal ‘opt-in’ discussions instead of the intended opt-out approach.

The neoGASTRIC trial is the largest individually randomised controlled trial enrolling preterm babies. Involving >60 UK and Australian recruiting sites, this pragmatic unmasked parallel group trial evaluates whether *not* routinely measuring gastric residual volumes (stomach contents) in preterm infants reduces the time taken for a baby to reach full enteral feeds, without increasing necrotising enterocolitis. A feasibility study^10^ and previous neonatal trial^4^ informed the study design including verbal opt-out consent (table 1). As part of an embedded process evaluation, we explored parent and staff views on this opt-out model to inform trial recruitment, staff training and future neonatal trials.

**Table 1:** neoGASTRIC verbal opt-out consent process

Step	Process description
1	neoGASTRIC posters and/or banners displayed in prominent places regularly visited by parents (eg, parents’ room or expressing room).
2	Babies are eligible for inclusion up until milk feeds reach >15 mL/kg/day for more than 24 hours; the neoGASTRIC information leaflet is provided to parents/guardians before eligible babies are randomised, and parents have an opportunity to verbally opt out before the baby is automatically enrolled. There is no consent form.
3	After randomisation, parents/carers who do not wish to continue with the allocated trial pathway are asked for permission to complete data collection—if they agree to ongoing data collection this does not constitute a withdrawal, but a discontinuation of the allocated trial pathway. Parents who wish to withdraw from data collection and the allocated trial pathway can do so at any point after randomisation.

## Methods

### Study design

We undertook a mixed methods process evaluation (August 2023 to April 2024) in four recruiting neonatal units. We aimed to interview and receive questionnaires from parents/guardians and staff related to the first 20 babies eligible for the trial, including those who opted their baby out of the trial. We conducted focus groups (FG) with staff involved in recruitment and clinical care. KW (female, social scientist, PhD) used previous research,^10^ and patient and public involvement feedback to develop information materials, surveys and topic guides which were developed iteratively.^11^

Questionnaires (onlinesupplemental files 1  2), interview and FG topic guides (onlinesupplemental files 3  4) explored views on trial acceptability including recruitment and consent processes, and factors that may have informed parental decision-making.

### Recruitment, sampling and conduct

Parents/guardians were required to speak English (gestational age at birth less than 34^+0^ weeks^+days^ with a nasogastric or orogastric tube in place). Neonatal staff assessed eligibility and provided parents with the neoGASTRIC process evaluation study information. Parents were invited to complete a questionnaire before leaving the hospital and/or to register interest in participating in an interview with a University of Liverpool researcher within a month. KW, TM (female, social scientist, PhD) and EN (female, trial manager) asked staff to complete an online or paper questionnaire at the end of their first shift caring for the baby.

TM contacted the parents who had registered to take part in an interview, initially in sequential order, then sampled for site variance and parents who had opted out. TM arranged interviews via videoconference or telephone as per parent preference and sought consent for participation in the interview. TM emailed each site after 4–6 months of recruitment and invited staff to register interest in an online FG.

Based on previous studies,^10^ we anticipated interviewing 15–25 parents and conducting two to four FGs to reach information power,^12^,^14^ which considers factors including the study aims, sample specificity, use of theory and the quality of dialogue. We anticipated around 80 parent and staff questionnaires related to 20 babies per site. The Consolidated Criteria for Reporting Qualitative Research checklist^15^ was used to aid reporting.

### Data analysis

TM and KW conducted the analysis. Digital audio recordings were transcribed verbatim by a transcription company (UK Transcription, Brighton, UK), checked and anonymised before being imported into NVivo V.14 software, for organising and coding. Reflexive thematic analysis of qualitative data was interpretive and inductive.^13 14^ TM and KW met regularly to develop the coding framework. Questionnaire data were inputted into SPSS Version 27 and descriptive statistics were used.

## Results

A total of 85 parents (59 mothers, 26 fathers) completed a questionnaire. Of these, 7/85 (8%) opted out of their baby’s involvement in neoGASTRIC; opt-out rate in the wider trial at the same point (152/2043, 7.4%). 51 parents registered interest in an interview and recruitment was closed at the point of information power. TM interviewed 15 parents (12 mothers, 3 fathers) either online (n=12) or via telephone (n=3); none had opted their baby out of the trial and 14/15 also completed the questionnaire. A total of 149 staff questionnaires were received relating to 96 babies across four sites. Of these, matched questionnaires from a parent and staff member(s) related to 63 babies. TM and KW conducted two FGs involving 18 nurses from three sites. Interviews lasted between 29 and 66 min (mean, 50 min). FGs lasted 65 and 46 min (mean 55 min). Table 2 shows the participant characteristics.

**Table 2:** Participant characteristics

Data collection method	Gender n (%*)	Role n (%)
Parent questionnaires (n=85)	Female 59 (69) Male 26 (30)	Mother 59 (69) Father 26 (37)
Parent interviews (n=15)	Female 12 (80) Male 3 (20)	Mother 12 (80) Father 3 (20)
Staff questionnaires (n=149)	Female 123 (98) Male 1 (1) Non-binary 1 (1) Missing 24 (16)	Student nurse 2 (2) Nursery nurse 3 (2) Associate practitioner 2 (2) Staff nurse 103 (80) Senior nurse 17 (13) Senior doctor (specialty trainees 4–8) 1 (1) Consultant 1 (1) Missing 20 (13)
Staff focus group 1 (n=3) (from sites 3 and 4)	Female 3 (100)	Staff nurse 3 (100), including the 2 research nurses
Staff focus group 2 (n=15) (from site 1)	Female 15 (100)	Staff nurse 14 (93), including the 2 research nurses Senior nurse 1 (7)

% may not equal 100 due to rounding

Synthesised findings from qualitative and quantitative data analyses are presented below. Illustrative quotations by qualitative themes and subthemes are shown in tables3^5^ and onlinesupplemental files 5  6.

**Table 3:** Theme: variance in how the verbal opt-out process was operationalised

Subtheme	Example quotes from parents	Example quotes from staff
Timing of approach in relation to randomisation	*“I believe somebody did mention something beforehand.”* (P2, mother, interview) *“Yeah, probably, probably were [randomised without discussion], especially if it’s the kind of retrospective or opt out consent… I definitely remember seeing the signs around and, I think in the back of my mind, I would probably enter that and then didn't really think much more of it.”* (P1, father, interview) Researcher: “*And was [Baby’s name] entered into the trial before you were aware of it?”* Mother: “*I don't remember.”* (P3, interview)	*“So, generally speaking, if they’re eligible, once I’ve given the leaflet, I will then randomise them, because it is opt-out.”* (S1, female, staff focus group 1) *“We always approach the parents first.”* (S2, female, staff focus group 1)
Timing of approach in proximity to labour and delivery	*“In the middle of labour.”* (P14, mother, interview) *“I hadn’t yet given birth when we were first spoken to about it.”* (P11, mother, interview) *“I was asked about joining these, like, literally a few hours after giving birth, and I’d had… is it pethidine, or something?”* (P15, mother, interview) *“They were on it within like the first hour [after giving birth]!”* (P2, mother, interview)	

**Table 4:** Theme: the potential burden of approaching parents too early

Example quotes from parents	Example quotes from staff
*“She was fantastic. She explained everything, all of the benefits and cons, what it was all about and everything like that. But I think I was probably, I don’t know, 13 hours through labour, maybe, by then, epidural and hadn’t eaten and drunk anything… I honestly can’t remember being given any leaflets. I think because it was a blur, really. (Laughter) I don’t know if that might’ve been passed to my husband and not myself, and put immediately into a bag or something. But yeah, I can’t recall if we were given anything. But we probably were, I don’t know.”* (P14 mother, interview) *“It is all a bit of a blur! They literally came straight in not long after I’d had him and I was like… of course I was still high—Entonox, gotta love it! [Laughs]. My poor little brain, just… Medically, I was fine. My brain was like, ‘Hell, no!’”* (P2, mother, interview) *“Oh my God, that’s quick to start agreeing to things for them when you’re not even over the shock that he’s even here [prematurely]… my brain was everywhere.”* (P13, mother, interview) *“It’s just a lot. Because obviously, you’re getting given so much information, it’s [the trial is] just another thing that you just have to think about. So, you just feel a bit overwhelmed.”* (P10, mother, interview)	*“I think we approach them quite early, so it’s all just a blur still.”* (S2, female, staff focus group 1) *“If I feel that maybe a parent is not taking it in, then I can make a decision as to whether I feel that it’s right to go back to them. Ultimately, if they’ve had the leaflet, and I’ve explained the study to them, then it is their responsibility, but I think some parents, who maybe do look like they’ve really not taken it in, I will go back to.”* (S1, female, staff focus group 1) *“The mum was really sick on delivery suite, and the girls had to go over with the information leaflet to try and see if the baby could go in it, but then they wanted to feed the baby quicker. So, you’ve only got a short window to go and do it.”* (S15, female, staff focus group 2) *“Didn’t understand. Approached too early. Parents initially opted out as felt overwhelmed but once it was explained more clearly, went back in.”* (SQ18, female, staff questionnaire)

**Table 5:** Perceptions of the neoGASTRIC consent process

Subtheme	Example quotes from parents	Example quotes from staff
Confusion about the use of the term ‘opt-out’	Mother: “*So, they came in pretty much straightaway once I’d been wheeled out of surgery to ask if we would do it [neoGASTRIC] and said that we needed to make a decision there and then because it needs to be decided quite quickly.”* Researcher: “*Did the nurse or doctor explain opt-out consent to you?”* Mother: “*No, I thought we-opted in.”* (P5, interview)	*“I think that there has sometimes been a bit of confusion around whether, for opt-out, the baby can be randomised before information is given, which is why I checked the protocol, to check and see.”* (S1, female, staff focus group 1) *“I think it’s strange because you’re saying that it’s not consented, but then they don’t get randomised until parents have had the information. So, you’re saying they don’t need to consent, but obviously they need the information. That’s fair enough. But then, at that point, they could choose whether to opt in or out of it, but they don’t need [to] consent. It’s just a bit confusing.”* (S4, female, staff focus group 2)
A decision to be made	*“The doctor was stood there waiting for us to give an answer, so it did feel a little bit like we couldn’t have a private discussion about it, in front of her. I remember that. But we both said how excellent she was at explaining it, and it felt like a no-brainer to us. But I could see, potentially, some pressure on parents, if the doctor’s not like, ‘I’ll give you five minutes to discuss and come back’.”* (P14, mother, interview) *“I just didn’t feel quite in the place to be making any sort of decisions, at that point.”* (P15, mother, interview)	

### Variance in how the verbal opt-out process was operationalised

Parents reported that they were first approached by neonatal staff about neoGASTRIC at varied times from immediately before, or during birth, to a few days after birth (table 3). Some mothers were approached during labour, when *“lying on your back with your legs akimbo*” (P14, mother, interview), or while *“waiting for a caesarean*” (P11, mother, interview). More commonly, mothers and partners (if present) were approached within hours or days of birth. One mother had no recollection of the timing of approach.

The majority of neoGASTRIC research discussions took place in a neonatal unit before randomisation, as per the process outlined in table 1. Parents who did not recall a discussion were not concerned about their child being entered into the trial without a research discussion, with one father describing how he had seen the poster and presumed his child would be involved.

Most staff stated they *“approach the parents first*” (FG1) with information before randomisation, yet some indicated that the mention of automatic randomisation in the protocol meant that it was unclear whether babies could be randomised “*before information is given, which is why I checked the protocol…. I think that that made me slightly nervous, that the protocol maybe wasn’t always being followed*” (S1, female, staff FG1).

### The potential burden of approaching parents too early

Questionnaire responses indicated that 70/85 (82%) of parents felt they were approached about the study at a convenient time. However, parent interviews, staff questionnaires and FGs indicated that parents approached during labour or shortly after birth were surprised at being asked to consider research and decide about their child’s involvement at such a difficult time. Some parents felt overwhelmed with information about their baby’s condition and felt they lacked capacity to consider research at that time (table 4). Some questioned the appropriateness of being asked about research when their child was critically unwell: *“I thought it’d be more of a priority to make sure he was, like, sort of, stable, before they’d ask those types of questions”* (P12, father, interview).

Staff in one FG described having to approach mothers early, when the timing may not be appropriate, because the baby was advancing feeds quickly, resulting in a shorter eligibility window. Recommendations from parents who felt they were approached too early included broaching the study before childbirth in the antenatal setting, so they could “*concentrate on what they’re*” being told (P13, mother, interview) and “*digest”* the trial information (P9, mother, interview). Parents who felt they were approached at a convenient time (eg, within days of birth rather than hours) appeared to have better recall of the discussion and felt the timing was appropriate when “*I was a little bit more with it”* (P7, mother, interview): *“By speaking to us on day 3 meant I could understand and support the study”* (P61, mother, questionnaire).

Despite large (>180 cm) study banners (see online supplemental file 7) and posters placed in prominent positions, only 18/85 (21%) of parents reported seeing the poster/banner, highlighting the importance of an appropriately timed research discussion.

### Perceptions of the ‘opt-out’ process

In FGs, staff described how the neoGASTRIC consent process was confusing (table 5). Some felt that approaching parents and providing information prior to randomisation was like an opt-in approach as “*at that point, they could choose whether to opt-in or out of it, but they don’t need consent. It’s just a bit confusing… I don’t feel it is an opt-out study”* (S1, female, staff FG2). One member of staff recalled one father questioning whether the consent process was truly an opt-out approach, because parents were still being asked to decide about their baby’s participation:

The dad, he did research and he was like… well, it’s an opt-out, but you’re giving me all this information and asking me if that’s okay. (S9, female, staff FG2)

Some mothers described how they were approached ‘pretty much straightaway’ after birth and felt pressured to decide about their baby’s participation ‘there and then’ which was interpreted as an opt-in decision as the opt-out process was not communicated.

### Support for opt-out consent for neonatal trials involving low-risk neonatal interventions

Staff were asked ‘how acceptable is the use of opt-out consent’ in the questionnaire; most indicated it was acceptable (75/121, 50.3%) or very acceptable (44/121, 36%); most also answered that they were satisfied with how they received neoGASTRIC information (72/85, 86%), and recalled receiving the written (80/85, 94%) or verbal study information from a doctor or nurse (80/85, 95%)—which they found to be clear and straightforward to understand (online supplemental file 8). Staff questionnaire data supported this: most stated that parents responded very positively (27/106, 26%) or positively (75/106, 71%) to their baby’s involvement in neoGASTRIC. The opt-out rate was low (7/85, 8%).

Mothers and fathers spoke of their support for verbal opt-out consent as the trial interventions (to measure or not measure gastric residual volumes) were not seen as invasive or posing a risk of harm for their child, as both were part of the usual UK clinical care (online supplemental file 5). Even parents who described the burden of being approached too early stated they did not opt their baby out, due to the perceived low-risk nature of the study and to help others in the future.

Parents approached with information and informed of automatic enrolment without a request to ‘make a decision’, stated it reduced the pressure of having to decide. Many implied they felt their rights to make an informed choice were protected as they could decide to opt out of the trial at any time. Some were reassured that they could change their mind and withdraw or ‘opt back in’. Staff echoed these views, often describing how an opt-out approach with a flexible window for eligibility reduces the potential burden of decision-making for parents when their child was critically ill.

### Reasons for opting out

Parents who opted their baby out stated that they did so due to uncertainties about the intervention and potential impact on their child, or use of data. None raised concerns about full informed consent not being sought. Staff data confirmed this, although a minority reported parents had opted out because they were approached too early (see online supplemental file 6 and table 4).

## Discussion

This is the first mixed methods evaluation to explore parent and practitioner experiences and views on verbal opt-out consent within a large multicentre neonatal trial. We found that parents and staff supported this approach to opt-out consent as they viewed the non-pharmaceutical intervention (no routine measurement of gastric residual volumes) as non-invasive and low risk. Some parents stated this approach reduced the burden of decision-making when their newborn baby was critically ill. Opt-out rates were low in our sample, but were representative of the wider trial, suggesting that the opt-out approach may support research inclusion by reducing bias in selecting who is approached.^16^

The validity of seeking informed consent from new parents whose baby is critically unwell in neonatal intensive care has been questioned.^17 18^ Opt-out consent in neonatal comparative effectiveness trials^4 8^ aims to reduce the potential burden of research discussions. Nevertheless, alternatives to informed consent, such as an opt-out approach, are not recognised in some countries; the ethics of any alternative to ‘opt-in’ consent have been debated; and there is inconsistency in how Research Ethics Committees view an opt-out model.^8^

As reported by McLeish *et al*,^9^ we found differences in how opt-out consent was operationalised by staff and experienced by parents. The neoGASTRIC opt-out process included a requirement to provide parents with information, through a leaflet, poster and discussion before randomisation. Most parents reported receiving written or verbal information about the trial before their baby was randomised. Exceptions may be explained by staff confusion about whether information had to be provided to parents before randomisation could occur, or by parents not recollecting information provided to them at a stressful time.^19^

There was wide variance in the timing of study information provision ranging from antenatal, during labour, to hours or days after birth. Women and partners approached within days of birth had good study recall and felt they had made an informed decision about their child’s involvement without the pressure of having to decide whether or not to opt out. These parents did not appear to view this opt-out approach as compromising their individual right to make an informed decision about participation, as they could opt out before randomisation. Parents viewed the process as ongoing, allowing them to change their mind to withdraw or ‘opt back in’.^9^

Accounts of parents approached during labour or within hours of birth suggested they felt overwhelmed and may have lacked the capacity to consider research, which is consistent with previous research around parents approached for opt-in consent with signed consent forms.^19^ Some staff operationalised an opt-in approach,^9^ requesting parents make an immediate decision before randomisation. Although none of these parents opted their baby out of the trial, their accounts suggested poorly timed discussions caused distress. Parents who opted their babies out stated that they did so due to uncertainties about how the allocated trial arm would impact their baby or the use of their baby’s data; no concerns were raised about automatic enrolment or not signing a consent form. Staff FGs and questionnaires indicated that some found the process confusing, and some research discussions were poorly timed and may have led to parents opting their baby out. Our findings have informed staff training and guidance for ongoing recruitment in neoGASTRIC (see online supplemental file 9).

Only 21% of parent questionnaire respondents saw the neoGASTRIC banners or posters despite prominent placement in units, highlighting the importance of verbal and written information sheets to inform decision-making.

Our findings support previous qualitative research in obstetric^20^ and paediatric trials^21^ on the practical and ethical complexities of conducting research in time-limited situations. Babies were eligible for inclusion in neoGASTRIC until they were receiving >15 mL/kg/day of milk. For premature babies, this often allowed several days to approach parents; however, in less preterm babies, more rapid increases in feeds meant that parents needed to be approached earlier. Parents suggested providing trial information at least a day after birth, or antenatally in the days preceding labour. Antenatal provision of trial information is challenging as preterm birth may not be predictable,^20^ and there are practical and ethical implications to consider as some babies may never become eligible to participate.

Future neonatal trials should consider whether RWPC, sometimes termed ‘deferred consent’, may be more appropriate if interventions are time critical. Literature has demonstrated staff and parental support for the use of RWPC in paediatric critical care,^10 21 22^ emergency department^23^ and obstetric trials^20^ involving interventions where urgent action is required for the purposes of the trial.^24^ Guidance^25^ is available for neonatal and paediatric critical care trials, yet rarely applied in the neonatal setting.

### Strengths and limitations

Findings are strengthened by a mixed methods design including site staff, mothers and birth partners who did, and did not, opt out of their baby’s involvement in the trial. Questionnaires were completed shortly after the trial was discussed and interviews were conducted within a month of recruitment to assist recall of recruitment and opt-out experiences. neoGASTRIC is an international trial with around 60 sites in the UK and Australia; however, this process evaluation was limited to four UK sites in the pilot phase. We had initially aimed to conduct the process evaluation over 4 months to gain insight into the recruitment of the first 20 babies. Recruitment was extended to 9 months as a larger questionnaire sample (85 parents and 149 staff) was needed to gain insight into why parents were opting out, to include a fourth site and fathers/birth partners in interviews. FGs were limited to nursing staff as medical staff did not respond to invitations. More nurses attended FGs from site 1 (a large unit) than sites 2 and 3. The parent interview sample was limited to parents who did not opt out and parents who could speak English.

## Conclusion

Parents and staff viewed the verbal opt-out approach to consent used in neoGASTRIC as acceptable and proportionate in low-risk, non-pharmaceutical, non-invasive, neonatal comparative effectiveness trials. Where interventions are time critical, RWPC should be considered.

## Supplementary material

10.1136/archdischild-2025-328693 Supplementary file 1

10.1136/archdischild-2025-328693 Supplementary file 2

10.1136/archdischild-2025-328693 Supplementary file 3

10.1136/archdischild-2025-328693 Supplementary file 4

10.1136/archdischild-2025-328693 Supplementary file 5

10.1136/archdischild-2025-328693 Supplementary file 6

10.1136/archdischild-2025-328693 Supplementary file 7

10.1136/archdischild-2025-328693 Supplementary file 8

10.1136/archdischild-2025-328693 Supplementary file 9

## Data Availability

No data are available.
