# Supplementary material for: Parent and practitioner experiences of opt-out consent in neonatal intensive care: a mixed methods study within a trial
Source: Arch Dis Child Fetal Neonatal Ed. 2025 Aug 31;111(2):e328693. doi: 10.1136/archdischild-2025-328693 (PMC13018813; doi:10.1136/archdischild-2025-328693)
Supplement: Supplementary file 1 [file fetalneonatal-111-2-s001.pdf]

Study number: ☐☐☐☐☐

neoGASTRIC

Parent/legal Representative Consent and Questionnaire

The following questions are about the neoGASTRIC study. We would like your views about the trial and how you were informed about your baby taking part in it. This questionnaire is part of an evaluation taking place in the first part of the trial to help find out whether any changes are needed to make the trial better.

**Please read each statement and make sure that you understand it. Once you have done this, if you agree with each statement, please write your initials in the box next to it.**	**Please initial box**
1. I confirm that I have read and understand the participant information sheet version v1.0 dated 06/12/2023 for the neoGASTRIC process evaluation study and have had the opportunity to ask questions which have been answered fully.	Initials
2. I consent to take part in the neoGASTRIC process evaluation study interview and complete this questionnaire.	Initials
3. I understand that my participation is voluntary, and I am free to withdraw at any time, without giving any reason and without my legal rights nor treatment / healthcare being affected.	Initials
4. I understand that brief quotations may be included in study reports and that nobody will be able to identify me in these reports.	Initials
5. I agree to data from my questionnaire responses being stored at the University of Liverpool for up to 10 years after the end of this study for checking purposes. I understand that these will be stored securely in compliance with the Data Protection guidelines.	Initials
6. I understand that the questionnaire may be looked at by responsible individuals from Imperial College London, or from regulatory authorities where it is relevant to my taking part in this research.	Initials
7. I give/do not give (delete as applicable) consent for information collected about me to be used to support other research by an academic institution or commercial company in the future, including those outside of the United Kingdom (which Imperial has ensured will keep this information secure).	Initials
8. I understand that data collected from me are a gift donated to Imperial College and that I will not personally benefit financially if this research leads to an invention and/or the successful development of a new test, medication treatment, product or service	Initials

Your full name: *(PRINT)* ____________________

Your Signature: *(For face to face only)* ____________________

Today's Date: D D / M M / Y Y

Study number: ☐☐☐☐☐

Today's date: D D / M M / Y Y

Are you the baby's Mother ☐ Father ☐ Other ☐

If Other, please specify: ______________________

Your baby's age (in days) ☐☐ *days*

Which hospital is your baby in? ______________________

What is the name of the unit your baby is in (e.g. NICU, SCBU, LNU):

____

Please provide the first part of your postcode
(e.g. If your postcode is L35 1AB, write L35): ☐☐☐☐

How would you describe your ethnic background? ______________________

What is your first language? ______________________

1. **How did you receive information (video) about the neoGASTRIC study?** *(please tick all that apply)*

   - The neoGASTRIC Study animation (video) for parents on a tablet device ☐
   - Parent Information sheet ☐
   - Poster ☐
   - Nurse or doctor explanation ☐
   - The neoGASTRIC study animation on the website ☐

2. **What language did you receive the study information in?** *(please tick all that apply)*

   English ☐ Welsh ☐ Bengali ☐ Polish ☐ Romanian ☐ Arabic ☐
   Urdu ☐ Panjabi ☐ Hindi ☐ Bulgarian ☐ Slovak ☐ Other ☐

   If Other, please specify: ______________________

3. **Please indicate how strongly you agree or disagree with the following statements by placing a circle around the answer that best fits your opinion or decision**

Statements	Agree	Unsure	Disagree
I saw The neoGASTRIC study poster	1	2	3
I watched The neoGASTRIC study video on a phone/tablet	1	2	3
A doctor or nurse discussed The neoGASTRIC study with me	1	2	3

Statements	Agree	Unsure	Disagree
I was given an information leaflet about The neoGASTRIC study	1	2	3
I was initially surprised to find out that my baby had already been entered into The neoGASTRIC study	1	2	3
The neoGASTRIC study information was provided to me at a convenient time	1	2	3
The information I received about The neoGASTRIC study was clear and straightforward to understand	1	2	3
I understand why written consent was not sought for my baby's participation in The neoGASTRIC study	1	2	3
I had enough opportunities to ask questions about The neoGASTRIC study	1	2	3
I was satisfied with how I was given information about The neoGASTRIC study	1	2	3
It was difficult to take in the information I was given about The neoGASTRIC study	1	2	3

4. Did you opt-out (say no) to the use of your baby's information or involvement in The neoGASTRIC study?

Yes ☐ *Go to question 5* No ☐ *Go to question 6*

5. If <u>you did</u> opt-out, it would be helpful to know your reasons for deciding that your baby's information would not be used, or they would not be taking part. Please could you provide these below, then *please go to question 7***.**

____

____

____

6. Why did you NOT opt-out of (why did you agree to) the use of your baby's information or involvement in The neoGASTRIC Study?

Please tick all that apply <u>and then circle your main reason</u> *(e.g.* ✓*)*

- To help my baby ☐
- To help other babies in the future ☐
- I felt that medical studies like The neoGASTRIC Study are important ☐
- Because I trusted the doctor or nurse who explained The neoGASTRIC Study ☐
- My baby no longer needs a gastric tube ☐
- I didn't feel comfortable saying no to the nurse or doctor who explained the study ☐
- Other ☐

If Other, please specify: ____________________

7. The following questions are about the decision you made for your child to remain in The neoGASTRIC study or to opt out of the study (whichever decision you made):

Do you feel sure about the best choice for your baby)? Yes ☐ No ☐

Do you know the benefits and risks of each option *(e.g. option of being in the trial, or opting out of the trial)*? Yes ☐ No ☐

Are you clear about which benefits and risks matter most to you/your baby? Yes ☐ No ☐

Do you have enough support and advice to make a choice? Yes ☐ No ☐

8. Do you have any concerns about The neoGASTRIC study? Yes ☐ No ☐

If Yes, please explain your concerns: ____________________

____

____

Please tell us any comments or suggestions you have to improve how we provide information to parents about The neoGASTRIC study:

____

____

____

____

We would be really interested in hearing more about your views and experiences of The neoGASTRIC study as part of the evaluation. If you agree to be contacted for a telephone/online interview at a later date, please provide your consent and details below:

Please read each statement and make sure that you understand it. Once you have done this, if you agree with each statement, please write your initials in the box next to it.	Please initial box
I give / do not give *(delete/mark as applicable)* give consent to being contacted about the possibility to take part in telephone/online interview.	*Initials*

Telephone number: ☐☐☐☐☐☐☐☐☐☐

Mobile number: ☐☐☐☐☐☐☐☐☐☐

Email address: ____________________

neoGASTRIC Study Team

NPEU Clinical Trials Unit, University of Oxford, Old Road Campus, Headington, Oxford, OX3 7LF.

01865 617927

neogastric@npeu.ox.ac.uk *www.npeu.ox.ac.uk/neogastric*

The neoGASTRIC study is funded by the National Institute for Health Research (NIHR) Health Technology Assessment programme (project reference NIHR134216)
The views expressed are those of the author(s) and not necessarily those of the NIHR or the Department of Health and Social Care
