# Supplementary material for: Parent and practitioner experiences of opt-out consent in neonatal intensive care: a mixed methods study within a trial
Source: Arch Dis Child Fetal Neonatal Ed. 2025 Aug 31;111(2):e328693. doi: 10.1136/archdischild-2025-328693 (PMC13018813; doi:10.1136/archdischild-2025-328693)
Supplement: Supplementary file 2 [file fetalneonatal-111-2-s002.pdf]

Study number: ☐☐☐☐☐

neoGASTRIC

Process Evaluation Questionnaire

The following questions are about the first 20 babies enrolled in The neoGASTRIC study at your site. We would appreciate your views on the experiences of recruitment and opt-out consent related to these babies, as well as any potential adherence issues and training needs. This questionnaire is part of an evaluation taking place in the first part of the trial to inform the ongoing trial. **Your responses will remain confidential.**

This questionnaire should be completed by **staff involved in the clinical care of babies** in centres taking part in The neoGASTRIC study. A member of **staff involved in the clinical care of each baby eligible to take part in The neoGASTRIC study should complete this questionnaire relating to their first shift caring for that baby**. Please complete a questionnaire for each of the first 20 babies eligible at your site. This includes babies whose parents opted out of The neoGASTRIC study.

Please note that by returning this survey you are giving permission for your responses to be included in The neoGASTRIC study process evaluation. All information will be anonymised and stored securely at The University of Liverpool in compliance with the Data Protection regulations.

Centre number: ☐☐☐☐☐

Baby trial number *(if applicable)*: ☐☐☐☐☐

Today's date: D D / M M / Y Y

Your role: Junior Nurse (band 5-6) ☐ Senior Nurse (band 7-8) ☐ Junior Doctor (ST1-3) ☐ Senior Doctor (ST4-8) ☐ Consultant ☐ Dietician ☐

Other *(please specify)* ____________________

Your gender: Male ☐ Female ☐

Which neoGASTRIC study arm was this baby allocated to? *(please tick only one)*:

No routine measurement of gastric residual volume NO GRV ☐

Routine up to 6 hourly measurement of gastric residual volumes ROUTINE GRV ☐

Recruitment and consent

1. **How did parents receive information about the neoGASTRIC study?** *(please tick all that apply)*
   - Animation on a tablet on the neonatal unit ☐
   - Information sheet ☐
   - Nurse or doctor explanation ☐
   - Link provided to access the animation on the study website ☐
   - Not provided at this point in time ☐

2. **Is there a neoGASTRIC study poster displayed in your unit?** Yes ☐ No ☐

3. **What language was the trial information provided to parents in?** *(please tick all that apply)*

   English ☐ Welsh ☐ Bengali ☐ Polish ☐ Romanian ☐ Arabic ☐
   Urdu ☐ Panjabi ☐ Hindi ☐ Bulgarian ☐ Slovak ☐ Other ☐

   If Other, please specify: ____________________

4. **Did the parents ask any questions about The neoGASTRIC study?** Yes ☐ No ☐

   If Yes, please outline what questions they had: ____________________

5. **How have parents responded to their baby's involvement in The neoGASTRIC study?**

   Very positively ☐ Positively ☐ Negatively ☐ Very negatively ☐ Not applicable ☐

   Please explain your answer: ____________________

6. **Did the parents of this child opt out of The neoGASTRIC study?** Yes ☐ No ☐

   If Yes, please any reasons provided by parents who opted out of their baby's involvement in The neoGASTRIC study? ____________________

7. **How acceptable did you find the use of opt-out consent in The neoGASTRIC study?**

   Very acceptable ☐ Acceptable ☐ Not acceptable ☐ Very unacceptable ☐

8. **Please tell us any suggestions you have for improving the recruitment process for The neoGASTRIC study, or any further comments about it:**

   ____
   ____
   ____
   ____
   ____
   ____

Process and training

9. **Have you measured GRV for this baby during this shift?** Yes ☐ No ☐

If this baby has been allocated to the 'no routine gastric residual volume measurement' arm of the trial and you have measured gastric residual volumes, please state the reason why you have measured gastric residual volumes GRV for this baby:

____

____

____

10. **Have you experienced any difficulties in adhering to the protocol in relation to this patient?** Yes ☐ No ☐

If Yes, please elaborate:

____

____

____

____

11. **How acceptable do /would you find not measuring GRV in babies enrolled in The neoGASTRIC study (i.e. not just this baby)?**

Very acceptable ☐ Acceptable ☐ Not acceptable ☐ Very unacceptable ☐

12. **How acceptable do you/would you find measuring GRV in babies enrolled in The neoGASTRIC study (i.e. not just this baby)?**

Very acceptable ☐ Acceptable ☐ Not acceptable ☐ Very unacceptable ☐

13. **Do you think The neoGASTRIC study site training could be improved?** Yes ☐ No ☐

If Yes, please elaborate:

____

____

____

____

Thank you for taking the time to complete this questionnaire. Please place in a stamped addressed envelope and return to The University of Liverpool team

(or press submit if online version)

neoGASTRIC Study Team

NPEU Clinical Trials Unit, University of Oxford, Old Road Campus, Headington, Oxford, OX3 7LF.

01865 617927

neogastric@npeu.ox.ac.uk *www.npeu.ox.ac.uk/neogastric*

NHMRC-NIHR Collaborative Research Grant Scheme. The views expressed are those of the author(s) and not necessarily those of the NIHR, NHMRC or the Department of Health and Social Care.
