# Supplementary material for: Parent and practitioner experiences of opt-out consent in neonatal intensive care: a mixed methods study within a trial
Source: Arch Dis Child Fetal Neonatal Ed. 2025 Aug 31;111(2):e328693. doi: 10.1136/archdischild-2025-328693 (PMC13018813; doi:10.1136/archdischild-2025-328693)
Supplement: Supplementary file 3 [file fetalneonatal-111-2-s003.docx]

**Sections of process evaluation topic guide related to recruitment and consent**

**Please note: *Italic text indicates instruction for researcher and will not be read to participant. Only sections of questions relevant to this paper are included.***

**Introductions**

***Check consent for audio recording – press record***

**Role**

1. Please can you go around the room and introduce yourselves, your role in neoGASTRIC and where you work?
2. How long have you been involved in clinical trials?
3. Before neoGASTRIC began, did you routinely measure GRV?
4. *If yes,* please can you explain your reasons for routinely measuring GRV? *(Prompt, explore if there was unit guidance on GRV measurement)*
5. *If no,* please can you explain your reasons for not routinely measuring GRV?
6. Is your usual practice different to that of the unit where you work?

**Information giving and consent process**

1. When do you check eligibility?
2. At what time point do you usually randomise after a baby meets the inclusion criteria?
3. Are the neoGASTRIC posters/banners placed in your unit? *(Prompt - explore where)*
4. When do you typically approach parents? *(Prompts - before or after randomisation?; explore how many hours)*
5. Are parents surprised to be asked about research at that point in time?
6. How do parents respond to being approached about the neoGASTRIC trial?
7. What sort of questions do parents typically ask?
8. Do you explain what Gastric Residual Volumes are to parents or is other terminology used? (*If other terminology, e.g. aspirates, ask,* What terminology do you use with parents? (*establish how the difference for measuring pH and GRV is explained*). Do you think that parents understand what GRV are?
9. Do you see or envisage any potential risks or benefits to babies in participating in the neoGASTRIC study? Do you describe these to parents?
10. Have you ever entered a baby into the trial before a research discussion with parents? *(Prompt: explore timeframe of randomisation and then parents being aware if known)*
11. When do you think is the best time to approach parents about the neoGASTRIC study?
12. Please tell me how you explain the neoGASTRIC study to parents *Explore what wording they use/if parents have any knowledge about the trial before approach, such as posters.*
13. How long do parents tend to get to think about whether they want their baby to take part in the neoGASTRIC trial or not *(prompt, explore time for opt-out)*?
14. Do you think this is long enough? / How long do you think parents should be given to think about taking part in a trial like this?
15. Do you explain opt out consent to parents?
16. Have you previously been involved in a trial that has used opt out consent? If yes, was the process similar to the neoGASTRIC process? Did those trials suggest you speak to parents before randomisation? *(Explanation if needed: all eligible babies are automatically included in the neoGASTRIC study if they are in a unit that is taking part in this trial, and parents are informed about the study through posters, leaflets, and sometimes an animation and discussion with the research team. Parents can ‘opt out’ of their babies’ involvement in the trial at any point. Information collected up until the point that parents ‘opt out’ are included in the study and there is not a consent form to sign –* **Is this the process that you have followed?***)*
17. What do you think about the use of opt out consent in the neoGASTRIC study (e.g. all eligible babies are included, and parents can withdraw from the trial and data collection)?
18. Do parents raise any concerns about the use of opt-out consent with you

**Training**

1. Who provides training at your site?
2. Are there any refresher sessions for staff?
3. Do you have any suggestions for improving neoGASTRIC study training at your site?

Before we finish today, is there anything else that you would like to say about the neoGASTRIC trial?

Thank you for your time today.
