# Supplementary material for: Parent and practitioner experiences of opt-out consent in neonatal intensive care: a mixed methods study within a trial
Source: Arch Dis Child Fetal Neonatal Ed. 2025 Aug 31;111(2):e328693. doi: 10.1136/archdischild-2025-328693 (PMC13018813; doi:10.1136/archdischild-2025-328693)
Supplement: Supplementary file 4 [file fetalneonatal-111-2-s004.docx]

**Sections of process evaluation topic guide related to recruitment and consent**

**Please note: *Italic text indicates instruction for researcher and will not be read to participant. Only sections of questions relevant to this paper are included.***

I will start with some questions about you if that’s ok and then I will ask you about your experience of the neoGASTRIC study. Is that ok?

| **DEMOGRAPHIC INFORMATION**  Do you mind if I start by asking a few questions about you and your child, for administrative purposes. | |
| --- | --- |
| 1.1 | Where do you live/What is the first part of your postcode? |
| 1.2 | Would you describe yourself as being: employed or unemployed?  - (If employed, what is your profession?) |
| 1.3 | How old is your baby/what is their DOB? Boy or girl if not mentioned already? What was their gestation? |
| 1.4 | What would you describe as being your first language and ethnicity? |
| 1.5 | So, just to get a background of what happened: what led to your baby needing intensive care?  Did your baby need ventilation? (invasive or CPAP?) |
| 1.6 | Did the doctor give your baby a diagnosis (e.g. did they tell you what had caused the need for care?) |
| 1.7 | How long ago was this? *Request month and date.*  Are you still in hospital/How long were you in hospital for?  How is [baby name] now? Has he/she recovered? |
| **THE NEOGASTRIC STUDY CONSENT PROCESS – BASELINE KNOWLEDGE** | |
| **2.1** | Would you mind if I start by getting an overall picture of what happened when you first heard about the neoGASTRIC study… could you tell me a bit about that?  *Explore any knowledge about the trial before approach, such as posters. Explore where parents were when they first heard the trial mentioned.* |
| **2.2** | Did you see any leaflets or posters about the study *(if not already stated)*?  *If yes,* Where were the posters or leaflets? *(Prompt: on the wall in PICU)*  Were you pointed towards the posters or leaflets at all? Could you tell me what you thought about the leaflets/posters?  Was there anything that you found: a) unclear? b) surprising?  What language did you receive the study information in?  Could the information leaflet be improved in any way? *(Prompt: If so, how?)* |
| **2.3** | Parents from some hospital sites were shown an animation of the NeoGASTRIC study information on a tablet.  ***For parents from sites who did NOT show the animation on a tablet device in NICU, say:***  The neonatal unit of your baby’s hospital site did not show the animation on a tablet device. Would it be OK if I show you the animation at the end of this interview and ask you some questions about it?  ***For parents from sites who used animation on tablet device in NICU, ask the following questions:***  The neonatal unit of your baby’s hospital site did show parents the animation on a tablet device. Do you remember seeing this? ***If no, ask*:** Would it be OK if I show you the animation at the end of this interview and ask you some questions about it?  ***FOR PARENTS WHO SAW THE ANIMATION, ASK:***  What did you think of the format of the study information using an animation on a tablet?  Was there anything that you found: a) unclear? b) surprising?  How did you feel when you were shown the animation?  Could the animation be improved in any way? *(Prompt: If so, how?)* |
| **2.4** | Did a member of staff discuss the trial with you? *(Explore initial reactions if before).*  **If Yes:** Can you recall whether it was a doctor or a nurse who spoke to you about the trial?  Can you tell me what they explained about the neoGASTRIC study?  Approximately how many hours after your child was born did they approach you?  Did they check with you that it was a good time to talk about research?   - *If so,* When was this? Do you think that this was the best time? - *If not,* When would have been the best time?   Were you surprised to be asked about research at that point in time?  Did one of the nursing staff looking after your baby introduce you to the research nurse or doctor? *Prompt:*   - Did you have an opportunity to discuss your child’s condition with a member of the clinical team? *Explore preferences for this, or just research staff* - Do you think it should be a doctor or nurse involved in a child’s care who approaches parents about a trial? Do you think it should be someone separate from the care team? - How could this be improved? |
| **2.5** | Do you think a member of staff should discuss the trial with parents, or is a leaflet, poster or animation enough to inform your decision?  Could you tell me why you think this? |
| **2.6** | This is a question I ask all parents and it’s not a test, but just so we can gauge whether the trial is being explained clearly enough.  Please can you tell me what the neoGASTRIC study was looking at?  Please describe your understanding of what the neoGASTRIC study is aiming to do? |
| **2.7** | Do you know what Gastric Residual Volumes are? *If not explain:* This is when the baby’s stomach contents are emptied (using a syringe to gently aspirate or suck the stomach contents through a port in your baby’s feeding tube) and measured before the next feed.  Does this change your view on the study at all?  Do you think this is important information? Should what GRV is be clarified?  Is there anything about how the neoGASTRIC study was explained to you that could have been handled a bit differently? |
| **2.8** | Were you provided with information about any of the potential risks or benefits of your baby taking part in the trial at that point? *If yes,* how did they describe these?  Was there anything that you found: a) unclear b) surprising?  Is there anything else that sticks out in your mind about the discussion? |
| **OPT OUT CONSENT AND SAMPLE COLLECTION** | |
| **4.1** | *Explain:* As both care pathways are used in the care of neonates across the UK, all eligible babies are automatically included in the neoGASTRIC study if they are in a unit that is taking part in this trial, and parents are informed about the study through posters, leaflets, and sometimes an animation and discussion with the research team. Parents can ‘opt out’ of their babies’ involvement in the trial at any point. Information collected up until the point that parents ‘opt out’ are included in the study and there is not a consent form to sign. This is called ‘opt out consent’.  Did the nurse or doctor explain opt out consent to you? Was it explained clearly? |
| **4.2** | What do you think about the use of opt out consent in The neoGASTRIC study (e.g. all eligible babies are included, and parents can withdraw from the trial and data collection)? |
| **4.3** | Was your baby entered into the trial before you were aware of it? *(Prompt: explore timeframe of randomisation and then parents being aware if known)*  What did you think when you found out that your baby had automatically been entered into the trial?  Were you surprised at all? If YES, Could you tell me a bit about that? |
| **4.4** | Did you have any concerns about this method? If so, could you tell me a little bit more about these? If not, what were your reasons for this?  Did you raise these concerns with a practitioner? *If so*, were they addressed/alleviated and how? *If not,* why?  **IF in the NO GRV GROUP**: How would you have felt if your baby had been allocated to the routine care group of the trial without your consent? *Explore any concerns*  **IF IN THE ROUTINE CARE GROUP**: How would you have felt if your baby had been allocated to the NO GRV arm without your consent? *Explore any concerns* |
| **4.5** | Unless parents have opted out, the neoGASTRIC team will access health records to collect data on babies to see their progress, such as their weight and head circumference or to identify cases of things like Necrotising enterocolitis (also called NEC).  Were you aware these data would be collected? *If not,* do you think we need to clarify this? What is the best way to do that? |
| **4.6** | *IF in the NO GRV GROUP:* did you ever see a nurse or doctor measuring GRV? Did you discuss this with them? |
| **SECTION 5: DECISION-MAKING** | |
| **5.1** | Did you opt out of the NeoGASTRIC trial? |
| *Opt-outs* | |
| **5.2** | Was it difficult to say no? |
| **5.3** | Would you mind telling me your reason for saying no to your child’s involvement in the study? *(Explore concerns and if this could be addressed through information)* |
| **5.4** | Did you worry about how the doctor or nurse would respond? How did they respond? |
| *If remained in the trial* | |
| **5.5** | In making the decision about your child’s continued participation in the neoGASTRIC study, what sort of things went through your mind? |
| **5.6** | Thinking about the different types of information you received (list which ones out of: leaflet, poster, video, nurse or doctor description of the study), which one did you think informed your decision the most? Please explain why.  Which format did you prefer? Please explain why. |
| **5.7** | Some parents have said that it’s difficult to take in all the information about a research study when their child is ill.  Could you tell me about what it is like to have this information given to you and for you to think about it at what must have been a very difficult time? *(Prompt explore which format is most useful in this setting e.g. leaflet, poster, video, nurse or doctor description of the study)* |
| **5.8** | When do you think is the best time to approach parents about the neoGASTRIC study? |
| **5.9** | How long did you get to think about whether you wanted your baby’s information to be used in the neoGASTRIC study?  Do you think this was long enough/ How long do you think people should be given to think about taking part in a trial like this?  Could you describe the possible benefits you expected your child to gain from taking part in the neoGASTRIC study?  Did this influence your decision in any way?  (*Prompt:* Did taking part in research provide some distraction/foster a sense of control? *If so,* how? *If not,* did it increase stress levels?) |
| **5.10** | Did you see or envisage any possible risks to your baby in participating?  Did you have any concerns about your baby’s involvement in The neoGASTRIC study?  *If so,* what were these concerns?  Did you directly raise these concerns with a nurse or doctor?  *If so,* Were these concerns addressed / alleviated during this conversation?  What was most helpful about these conversations? |
| **5.11** | In making your decision, did you think about how the research may benefit other babies in the future? |
| **5.12** | Apart from the doctor or nurse, did you discuss it with anyone else? [Can you tell me a bit about that]? |
| **5.13** | Did you ever feel under pressure in making your mind up? [*If yes:* where did that pressure come from]? |
| **5.14** | How was the nurse/doctor in dealing with you that day? *Prompts:* how was their manor, how did they come across, were they empathetic, explained things clearly – not too leading? |
| **5.15** | In making your decision how important was their manor / their level of expertise / professionalism, such as what they said and how they said it? |
| **5.16** | Did you have the opportunity to ask any other questions about the trial? Have you thought of any questions you would have liked to have asked that you didn’t ask at the time? |
| **5.17** | Now that a little time has passed, how do you feel about the decision you made? |
| Before we finish today, is there anything else that you would like to say about the neoGASTRIC trial?  Thank you for your time today. | |
