# Supplementary material for: Parent and practitioner experiences of opt-out consent in neonatal intensive care: a mixed methods study within a trial
Source: Arch Dis Child Fetal Neonatal Ed. 2025 Aug 31;111(2):e328693. doi: 10.1136/archdischild-2025-328693 (PMC13018813; doi:10.1136/archdischild-2025-328693)
Supplement: Supplementary file 5 [file fetalneonatal-111-2-s005.docx]

| **Table 6. Theme: Support for opt-out for neonatal trials involving low risk interventions** | | |
| --- | --- | --- |
| **Sub theme** | **Example quotes from parents** | **Example quotes from staff** |
| For low-risk or non-invasive interventions | *“I don't think [I have any concerns about the use of opt-out consent]. As long as there's no harm in terms of what the study is about, as long as there's no risk for it, then I don't think it's an issue, personally”* (P10, mother, interview).  *“I think it’s [opt-out is] acceptable because it’s not so invasive”* (P4, mother, interview).  *“Because they're both [measuring GRV and not measuring GRV] valid methods already used… It can help inform future practice, fine for him to stay in… if it was like a more experimental one, then I’d have said, “Hang on. I'm not happy with this!”* (P9, mother, interview). | *“They [parents] weren't bothered either way. They said since it's routinely what we do and not invasive they were happy to continue with study”* (SQ20, female, staff questionnaire).  *“I like the opt-out… I quite like the opt-out consent”* (S2, female, staff focus group 1). |
| To reduce burden | *“It seems to have taken the pressure off by, you know, not having to decide”* (P2, mother, interview).  *“I think that’s ideal [opt-out consent]. That’s better for the parents as well, to be honest. They don’t feel forced. They don’t feel like it’s an obligation, if that makes any type of sense, then”* (P8, mother, interview).  *“I think because it’s such a shock to anybody… to have a… premature baby, … you’re not thinking straight. So, I think giving that option that you can actually opt out is beneficial, for somebody who thinks, “Oh, I’ve just said yes for the sake of it, at the time” just to sort of get somebody away from them, for the time being”* (P12, father, interview).  *“I think it's [opt-out consent is] the right thing to do… you know they'll [babies will] be getting fed anyway and they're going to be getting fed one of those two ways, depending on which area of the country they're in… So, I think yeah, it's the right way to do it and it just helps to get to that [research] answer quicker”* (P1, father, interview). | *“It must be difficult for the parents, you know, straight away, that somebody’s coming asking them all these questions, and I’m sure they don’t really know what day it is, never mind going into a trial. But I think probably the opt-out one, I think is probably better for the parents, you know, to come back at a later date, and opt out of it”* (S3, female, staff focus group 1).  *“With [another trial using opt out consent], you have to get it within three hours, and I find that it can be quite stressful, at times, like, it’s quite a- or it can be a panic to go do it, and I feel like it can get rushed, at times. So, I quite like how this one [neoGASTRIC] operates a bit better”* (S2, female, staff focus group 1). |
| Rights and choice | *“I know that you've got your rights if you wanted to opt out”* (P1, father, interview)  *“Having the opt out available is good, because parents’ choice can then be considered”* (P11, mother, interview)  *“I think it’s good that you can join a trial, and then you also have the option of withdrawing from it at any time. I think that’s reassuring. Because if you suddenly change your mind, or you realise you think it’s having an effect on your baby, you can still withdraw. I think that gives you that reassurance that you’re not tied into something for the whole time you’re in there, if you’re not comfortable with it”* (P15, mother, interview)  *“If you've got any doubts, just say no. Well, think and think. Keep reading things through and say, ‘Do we, don't we, do we, don't we?’ The decisions sort of made for you, so he is going to be in there. However, just ask them if you don't! It's probably just an easier way of doing it for parents”* (P2, Mother, interview) |  |
