# Supplementary material for: Parent and practitioner experiences of opt-out consent in neonatal intensive care: a mixed methods study within a trial
Source: Arch Dis Child Fetal Neonatal Ed. 2025 Aug 31;111(2):e328693. doi: 10.1136/archdischild-2025-328693 (PMC13018813; doi:10.1136/archdischild-2025-328693)
Supplement: Supplementary file 6 [file fetalneonatal-111-2-s006.docx]

| **Table 7: Theme: Reasons for opting out of neoGASTRIC** | |
| --- | --- |
| **Example quotes from parents** | **Example quotes from staff** |
| *“Because of discomfort for her and reduced feeds and agreed she had been through enough at the time”* (P54, mother, questionnaire)  *“We opted out, because of his heart. He needs to keep up the calories to gain weight, so we were like, “We can’t really risk with him missing feeds [due to being in the GRV arm] for a longer period of time”* (P7, mother, interview).  *“Just unsure if baby will receive a different type of care while at hospital”* (P13, mother, questionnaire).  *“I opted out because I didn't want to participate and I want my babies information confidential in every way”* (P10, mother, questionnaire).  *“Because I did not agree with video I watched. I want to opt out”* (P5, mother, questionnaire). | *“I think for our really extreme prems [premature babies], the parents, I think, are probably more concerned, and I think that also goes the same for the doctors are more concerned, with our extreme prems”* (S1, female, staff focus group 1).  *“Baby had worsening respiratory effort, mum emotional, withdrew from trial”* (SQ121, female, staff questionnaire).  *“Didn’t understand. Approached too early”* (SQ18, female, staff questionnaire).  *“Too early to decide”* (SQ86, female, staff questionnaire).  *“Have declined all research”* (SQ87, female, staff questionnaire). |
