# Supplementary material for: Parent and practitioner experiences of opt-out consent in neonatal intensive care: a mixed methods study within a trial
Source: Arch Dis Child Fetal Neonatal Ed. 2025 Aug 31;111(2):e328693. doi: 10.1136/archdischild-2025-328693 (PMC13018813; doi:10.1136/archdischild-2025-328693)
Supplement: Supplementary file 8 [file fetalneonatal-111-2-s008.docx]

| **Table 8. Parent questionnaire responses related to recruitment and consent decision making n=85*** | | | |
| --- | --- | --- | --- |
| **Statement** | **Agree**  *n (%)* | **Unsure**  *n (%)* | **Disagree**  *n (%)* |
| a. The doctor or nurse discussed the neoGASTRIC study with me | 80 (95) | 1 (1) | 3 (4) |
| b. I was given an information leaflet about the neoGASTRIC study | 80 (94) | 4 (5) | 1 (1) |
| c. I was initially surprised to find out that my baby had already been entered into the neoGASTRIC study | 20 (24) | 17 (20) | 48 (57) |
| d. The neoGASTRIC study information was presented at a convenient time | 70 (82) | 10 (12) | 5 (6) |
| e. The information I received was clear and straightforward to understand | 69 (81) | 13 (15) | 3 (4) |
| f. I understand why my written consent was not sought for my baby’s participation in the study | 63 (74) | 15 (18) | 7 (8) |
| g. I had enough opportunities to ask questions about the study | 77 (91) | 3 (4) | 5 (6) |
| h. I was satisfied with how I was given information about the study | 72 (86) | 9 (11) | 3 (4) |
| i. It was difficult to take in the information I was given about the study | 23 (27) | 16 (19) | 46 (54) |

~~*~~Statement a. 1/85 missing response, h. 1/85 missing response.
