# Supplementary material for: Parent and practitioner experiences of opt-out consent in neonatal intensive care: a mixed methods study within a trial
Source: Arch Dis Child Fetal Neonatal Ed. 2025 Aug 31;111(2):e328693. doi: 10.1136/archdischild-2025-328693 (PMC13018813; doi:10.1136/archdischild-2025-328693)
Supplement: Supplementary file 9 [file fetalneonatal-111-2-s009.docx]

**Guidance for ongoing neoGASTRIC training and recruitment**

1. Ensure neoGASTRIC posters and/or banners are displayed in prominent places.

2. Tailor for each family. It’s OK to pause randomisation to discuss the study and give parents more time to read about the neoGASTRIC trial.

3. All babies can be enrolled into neoGASTRIC up to 24 hours after they start feeding (feeds reach >15 ml/kg/day).

4. Do not approach parents during labour or too soon after birth; Provide trial information at least a day after birth, or antenatally in the days preceding labour.
